# When, Where, and Why Are Babies Dying? Neonatal Death Surveillance and Review in Bangladesh

**DOI:** 10.1371/journal.pone.0159388

**Published:** 2016-08-01

**Authors:** Abdul Halim, Juan Emmanuel Dewez, Animesh Biswas, Fazlur Rahman, Sarah White, Nynke van den Broek

**Affiliations:** 1 Centre for Injury Prevention and Research Bangladesh (CIPRB), Dhaka, Bangladesh; 2 Centre for Maternal and Newborn Health, Liverpool School of Tropical Medicine, Liverpool, United Kingdom; 3 Örebro University, Örebro, Sweden; Centre Hospitalier Universitaire Vaudois, FRANCE

## Abstract

**Background:**

Better data on cause of, and factors contributing to, neonatal deaths are needed to improve interventions aimed at reducing neonatal mortality in low- and middle-income countries.

**Methods:**

Community surveillance to identify all neonatal deaths across four districts in Bangladesh. Verbal autopsy for every fifth case and InterVA-4 used to assign likely cause of death.

**Findings:**

6748 neonatal deaths identified, giving a neonatal mortality rate of 24.4 per 1000 live births. Of these, 51.3% occurred in the community and 48.7% at or on the way to a health facility. Almost half (46.1%) occurred within 24 hours of birth with 83.6% of all deaths occurring in the first seven days of life. Birth asphyxia was the leading cause of death (43%), followed by infections (29.3%), and prematurity (22.2%). In 68.3% of cases, care had been provided at a health facility before death occurred. Care-seeking was significantly higher among mothers who were educated (RR 1.18, 95% CI: 1.04–1.35) or who delivered at a health facility (RR 1.48, 95% CI 1.37–1.60) and lower among mothers who had 2–4 previous births (RR 0.89, 95% CI 0.82–0.96), for baby girls (RR 0.87, 95% CI 0.80–0.93), and for low birth weight babies (RR 0.89, 95% CI 0.82–0.96).

**Interpretation:**

Most parents of neonates who died had accessed and received care from a qualified healthcare provider. To further reduce neonatal mortality, it is important that the quality of care provided, particularly skilled birth attendance, emergency obstetric care, and neonatal care during the first month of life is improved, such that it is timely, safe, and effective.

## Introduction

Every year, 2.9 million neonates die. These deaths happen mainly in low- and middle-income countries and most are considered to be preventable [1].

In 2014, the World Health Organization (WHO) and the United Nations Children's Fund (UNICEF) launched the Every Newborn Action Plan (ENAP), a road map to reduce preventable neonatal deaths [2]. One of the recommendations of the ENAP is improving the counting of every birth and neonatal death, raise awareness, and promote targeted actions to save lives. Moreover, the ENAP advocates the strengthening of maternal and neonatal death surveillance and response and support of community reporting and reviewing of maternal and neonatal deaths [2]. Indeed, identifying and understanding cause of, and circumstances preceding, death are crucial if the right intervention programmes are to be developed and implemented.

In many settings, community workers are in place and in principle surveillance of births and deaths is possible. Obtaining information to understand when and why a baby died can similarly be achieved by interviewing parents in the community using verbal autopsy (VA) methodology, as recommend by the WHO [3].

Bangladesh is often considered a champion in terms of health gains despite presenting poorer development indicators than other South Asian countries. This success is considered to be the result of the relatively high level of women empowerment, the implementation of an expanded network of community health workers and the presence of a multitude of stakeholders (including government and non-governmental organisations) providing healthcare [4]. Neonatal mortality has decreased from 55 to 27 per 1000 live births between 1990 and 2010 [5]. Notwithstanding this important reduction, the newborn mortality rate in Bangladesh has remained relatively high as in other low- and middle-income settings and currently represents two thirds of the under-five mortality rate [6].

Across many countries, the reduction in neonatal mortality needs to be accelerated in order to reach the ENAP target of less than 12 neonatal deaths per 1000 by 2030 [2]. Much better data and understanding of the cause of and factors contributing to neonatal deaths is needed overall; systems and processes for surveillance and monitoring over time are essential to do this. The development, implementation and evaluation of effective interventions and programmes aimed at reducing neonatal mortality such information is also vital.

The Government of Bangladesh implemented a population-based Perinatal Death Review (PDSR) system in four rural districts. We present the findings and an analysis of the information obtained via verbal autopsy to identify time and cause of death, contributing factors as well as the pattern of care seeking for ill neonates by parents in these districts.

## Methods

### Study area

Four target districts, (Thakurgaon, Jamalpur, Moulvibazar, and Narail) with a total population of 6.7 million, were chosen based upon their relatively poor maternal and neonatal health indicators: uptake of antenatal care (ANC) (63.5% *vs* 67.7% nationally), percentage of deliveries attended by a trained provider (19.9% *vs* 31.4% nationally) [7]. In addition, these districts are target districts of the joint Government and United Nations Maternal Newborn Health Initiative (MNHI) which focuses on saving maternal and neonatal lives through improved district level planning, investments in infrastructure and supplies and strengthening of human resources.

### Data collection tools

An expert team under the guidance of the Directorate General of Health Services (DGHS) and the Directorate General of Family Planning (DGFP) composed of neonatologists, obstetricians, health programme specialists and public health experts from the Centre for Injury Prevention, Health Development and Research Bangladesh (CIPRB) developed all study materials.

The verbal autopsy questionnaire was based upon the recommended WHO format and the existing neonatal death audit forms available in Bangladesh [3,8]. The questionnaire was adapted for use by district healthcare workers and family planning workers and translated into Bangla. The questionnaire was field tested in one district before being used in all four study districts. The questionnaire includes 39 closed questions with different response categories for socio-demographic characteristics of the family of the deceased, complications during pregnancy, antenatal care, and birth-preparedness, healthcare seeking behaviour at the time of neonatal illness, obstetrical and neonatal complications during delivery, and care seeking behaviour of parents as well as information on referral and delays.

### Data collection

All Health Assistants (HA) and Family Welfare Assistants (FWA) (community level workers responsible for a population of 5000–6000) were trained to identify each neonatal death that occurred in their area and complete a death notification slip.

All Health Inspectors (HI), Assistant Health Inspectors (AHI) and Family Planning Inspectors (FPI) (Field level supervisors of HAs and FWAs) who oversee a population of 25,000–30,000, received training in the conduct of a verbal autopsy for neonatal deaths. Each supervisor conducted of at least five verbal autopsies under supervision to ensure competency.

### Death notification

Trained HAs or FWAs used a network of local community members (teachers, community health workers and traditional birth attendants) to identify the death of any neonate in their assigned area. After being informed of a neonatal death, the HA/FWA visited the household to confirm the death as a neonatal death, defined as: “death of a neonate, i.e. a live birth born after 28 weeks of gestation and who showed any evidence of life, such as beating of the heart, pulsation of the umbilical cord, or definite movement of voluntary muscles, whether or not the umbilical cord has been cut or the placenta is attached, within 28 days after birth”. If the definition criteria were fulfilled, the HA/FWA completed a death notification form which was sent to an assigned focal person within 7 to 15 days following death.

### Verbal autopsy

**Fig 1:**
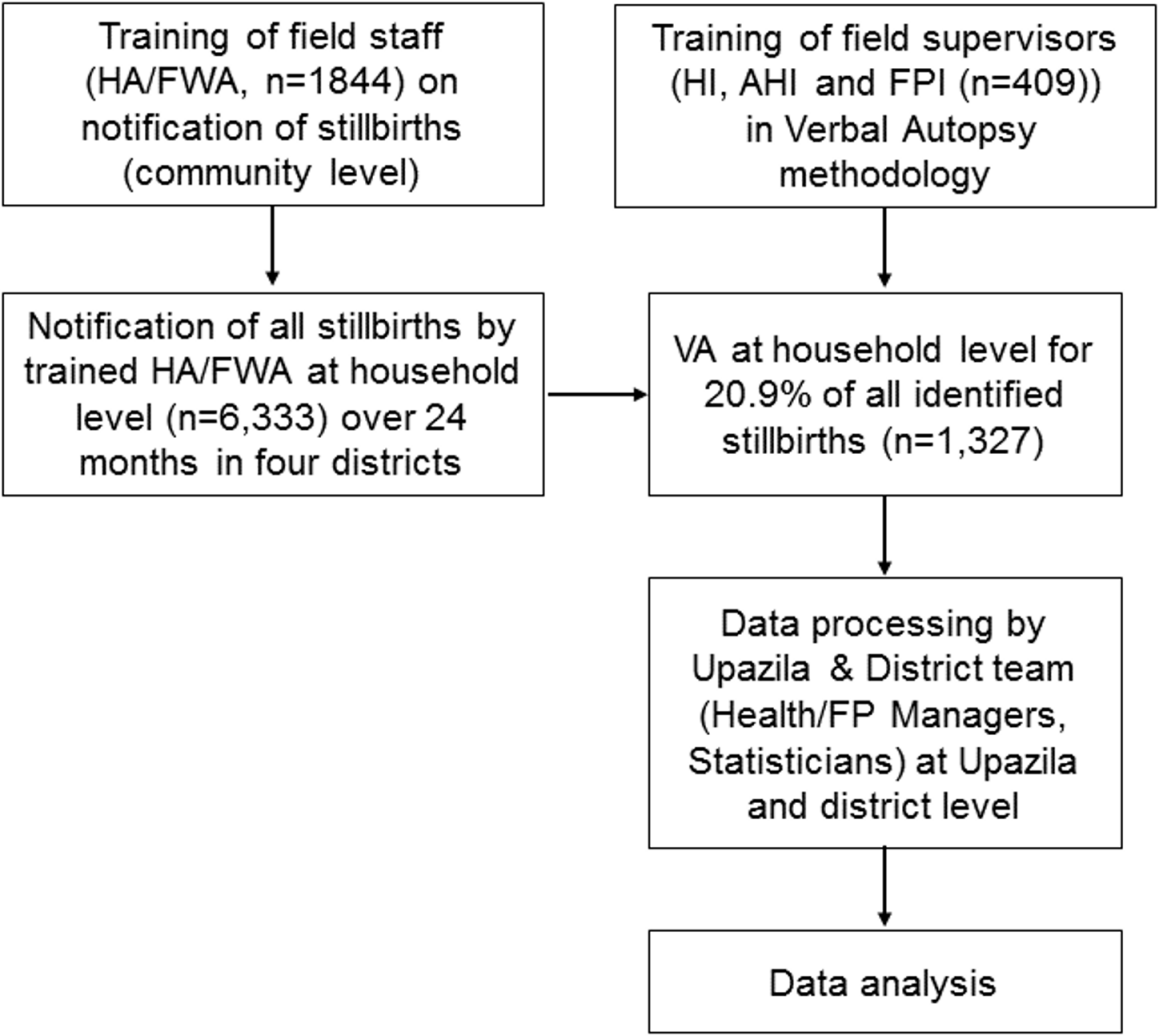
Neonatal death notification, verbal autopsy and reporting pathway.

Upon receipt of the death notification, a Trained Health (HI/AHI) or Family Planning Inspector (FPI) was assigned to conduct a verbal autopsy for every (consecutive) fifth reported neonatal death which occurred in his/her assigned area. Each verbal autopsy interview included the mother, father, or relatives who were present either at the birth or at the time of the death of the neonate (Fig 1).

### Data analysis

Socio-demographic characteristics were described using frequencies and proportions. Proportions for categorical variables were compared using chi-square test, at a significance level of <0.05.

Deaths which occurred at home and on the way to a health facility were treated as a single group (neonatal death at home) and compared with deaths that occurred at a health facility.

To assign cause of death, we used the computer-automated, probabilistic model InterVA-4 (version 4.02). This model determines the cause of death by processing successive indicators to generate up to three likely causes of death from 35 broad categories with an estimated probability for each assigned likely cause of death provided [9]. We selected the cause of death with the highest probability for use in further analyses. The verbal autopsy questionnaire included 39 (67%) of the 58 possible questions included in the InterVA-4 software package.

To identify determinants of care seeking, a binomial regression was used to derive risk ratios. Independent variables included in the univariate analysis were mother’s age, mother’s educational status, number of ANC visits, parity, reported complications during pregnancy, mode and place of delivery, sex of the neonate, neonatal birth weight, the presence of spontaneous breathing at birth, and the cause of neonatal death. Effect estimates were expressed as risk ratios with a 95% Wald confidence interval. All independent variables with statistical significance ≤10% (<0.1) in the univariate model were included in the full multivariable regression model. To obtain a parsimonious regression model, we examined multicollinearity between all the predictor variables by calculating the variable inflation factor (VIF). VIF threshold for multicollinearity was set at 4. Multicollinearity was not found. Finally, we used a backward elimination approach to retain only variables which were significant at the 5% level (p≤0.05).

Data entry and all analysis were performed using SPSS, version 22.

### Ethical approval

Ethical clearance was obtained from the Ethical Review Committee, Centre for Injury Prevention and Research Bangladesh (ERC/CIPRB/2010/01). The protocol and tools were reviewed and approved for implementation by the DGHS of Bangladesh. Informed written consent was requested and obtained from each respondent before each VA interview and consent forms are held by the Centre for Injury Prevention and Research Bangladesh. Anonymity and confidentiality was maintained throughout the process. Participation was voluntary.

## Results

A total of 6748 neonatal deaths were identified to have occurred over a 24 month period (from January 1^st^ 2011 to December 31^st^ 2012) in all four districts. Using the crude birth rate for Bangladesh (20.6 per 1000) [10], the total number of live births in the four districts was estimated at 276,040 giving a neonatal mortality rate in the four districts of 24.4 per 1000 live births, comparable to the national Bangladesh neonatal mortality rate of 24 per 1000 live births [11].

A total of 1433 verbal autopsies (21.2% of all deaths reported) were conducted. Of these, 735 (51.3%) of neonatal deaths occurred at home, 619 (43.2%) at a health facility, and 79 (5.5%) on the way to a health facility from home or during transfer between health facilities. The characteristics of mothers, deliveries, and neonates are presented in Table 1, disaggregated by place of death (home or health facility) and compared with general population data for Bangladesh, where available.

**Table 1:** Socio-demographic and obstetric characteristics of mothers, type of delivery and characteristics of neonates who died (n = 1,433).

	Demographic Health Survey Bangladesh 2011 (%)	Total deaths (n = 1,433)	Neonates who died at home (n = 814)	Neonates who died at a health facility (n = 619)
**1. Maternal characteristics**				
Median age of mother (years [Inter Quartile Range])		22 (20–26)	22 (20–26)	22 (20–25)
Mother’s education (n [%])				
Illiterate	27.7	225 (15.7%)	175 (21.5%)	50 (8.1%)
Primary education	30	511 (35.7%)	310 (38.1%)	201 (32.5%)
Secondary education	42.3	633 (44.2%)	313 (38.4%)	320 (51.7%)
Higher secondary and above		64 (4.4%)	16 (2.0%)	48 (7.7%)
Antenatal Care (index pregnancy) (n [%])				
- accessed ANC on at least one occasion	67.7	1173 (81.9%)	630 (77.4%)	543 (87.7%)
- accessed ANC four or more times	25.5	511 (35.7%)	24.5 (30.1%)	266 (43.0%)
Parity (n [%])				
1		803 (56.0%)	423 (52.0%)	380 (61.4%)
2–4		560 (39.1%)	345 (42.4%)	215 (34.7%)
5 or more		70 (4.9%)	46 (5.7%)	24 (3.9%)
**2. Delivery characteristics**				
Mode and place of delivery				
Vaginal delivery at home		872 (60.9%)	674 (82.8%)	198 (32.0%)
Vaginal delivery in a health facility		331 (23.1%)	91 (11.2%)	240 (38.8%)
Caesarean section		230 (16.0%)	49 (6.0%)	181 (29.2%)
Reported Complications during index delivery		711	352	359
Convulsions		68 (9.5%)	32 (9.1%)	36 (10%)
Haemorrhage		171 (24.1%)	102 (29.0%)	69 (19.2%)
Prolonged labour (>12 hour)		367 (52%)	165 (47%)	202 (56%)
Obstructed labour		236 (33.2%)	111 (31.5%)	125 (34.8%)
Premature rupture of membranes >24 hours		341 (48%)	157 (44.6%)	184 (51.3%)
**3. Neonatal characteristics**				
Gender				
Male		845 (59.0%)	458 (56.3%)	387 (62.5%)
Female		588 (41.0%)	356 (43.7%)	232 (37.5%)
Neonatal birth weight				
Smaller than normal		533 (37.2%)	358 (44.0%)	175 (28.3%)
Normal weight		723 (50.5%)	386 (47.4%)	337 (54.4%)
Bigger than normal		177 (12.4%)	70 (8.6%)	107 (17.3%)
Spontaneous crying or breathing at birth		775 (54.1%)	468 (57.5%)	307 (49.6%)

### Maternal characteristics

There was no difference in maternal age between neonates who died at home or at a health facility. The majority of mothers received at least primary education (84.3%). This was higher among the group of neonates who died at a health facility (91.9%) compared to those who died at home (78.5%) (p = <0.0001). For babies who died at a health facility, mothers were more likely to have attended antenatal care (87.7%), compared to the group who died at home (77.4%) (p = <0.0001) and were more likely to be primiparous (61.4% *vs* 52%) (p = 0.001).

### Delivery characteristics

Of the mothers of babies who had died, less than half had received skilled birth attendance at a health facility (561/1433, 39.1%) of which 41% (230/561) had been delivered by caesarean section. Fifty eight percent of deliveries were attended by a family member or a traditional birth attendant.

Almost half of mothers reported complications during delivery (711/1433) with prolonged or obstructed labour the most frequently reported followed by premature rupture of membranes (more than one complication can be reported in each case). The proportion of mothers reporting prolonged labour was higher for babies who died at a health facility compared to home (56.3% *vs* 46.9%, p = 0.012). For haemorrhage, this proportion was lower (19% *vs* 29%, p = 0.002).

Eighty three percent of neonates who died in the community were born in the community and 68% of those who died at a health facility were born in a health facility.

### Neonatal characteristics

More male than female neonates were reported to have died and included in the analysis (59% *vs* 41% respectively, p = 0.017). The proportion of babies reported to have spontaneously cried and/or starting breathing at birth was lower for babies who died at a health facility rather than at home (49.6% *vs* 57.5%, p = 0.003).

**Fig 2:**
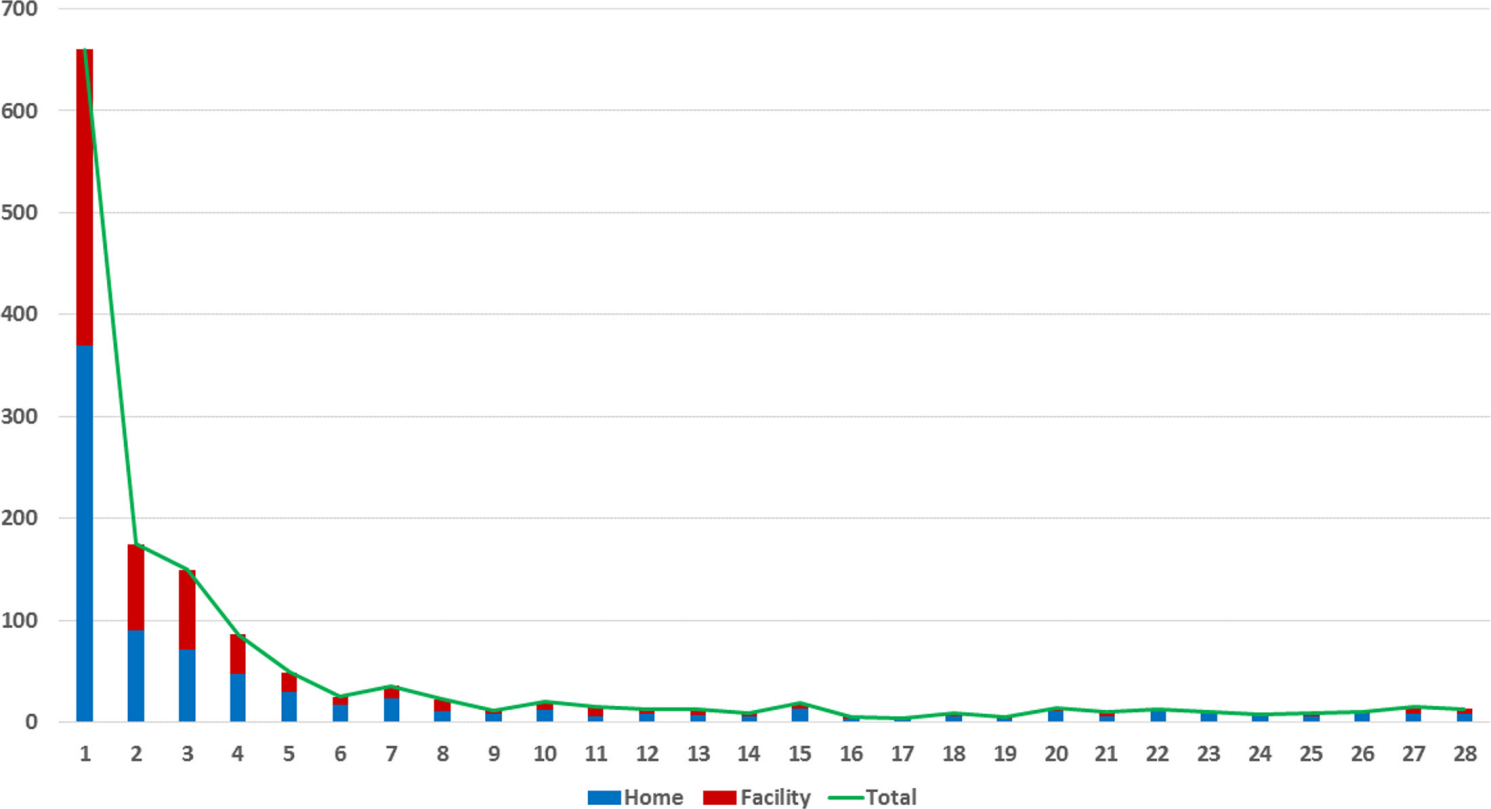
Age at time of death (in days) by place of death for early and late neonatal deaths (n = 1,344).

Thirty seven percent of the deaths were considered to be small for dates and these were more likely to have died at home than at a health facility (44% *vs* 28.3% respectively, p<0.0001). The distribution of age at time of death (in days) is provided in Fig 2; 46.1% died during the first 24 hours of life and of these 45.0% died at home and 46.8% at a health facility (p = 0.6). In total, 83.6% of all deaths occurred in the first week (0 to 6 days inclusive); early neonatal death) and 16.5% in the second to fourth week (between 7 and 28 days inclusive; late neonatal deaths).

### Cause of death

The InterVA-4 software provided at least one likely cause of death in 94.6% (1354) of all neonatal deaths for which verbal autopsy was conducted; with one cause assigned in 87.8%, two in 10.5% and three in 0.5% of cases (Table 2).

**Table 2:** First and second most probable cause of neonatal death assigned using InterVA probabilistic model (n = 1433).

	Most probable cause of death			Second most probably cause of death		
Cause of death	All deaths(1,433) n (%)	Neonatal death at home (814) n (%)	Neonatal death at a health facility (619) n (%)	All deaths(150) n (%)	Neonatal death at home(88) n (%)	Neonatal death at a health facility (62) n (%)
**Birth asphyxia**	**616 (43.0)**	326 (40.0)	290 (46.7)	**34 (22.7)**	19 (21.6)	15 (24.2)
**Neonatal pneumonia**	**342 (23.9)**	188 (23.1)	154 (24.9)	**50 (33.3)**	30 (34.1)	20 (32.3)
**Prematurity**	**318 (22.2)**	214 (26.3)	104 (16.8)	**47 (31.3)**	29 (32.9)	18 (29)
**Neonatal sepsis**	**74 (5.2)**	37 (4.5)	37 (6)	**4 (2.7))**	2 (2.3)	2 (3.2)
**Meningitis and encephalitis**	**3 (0.2)**	3 (0.4)	0 (0)	**-**	-	-
**Congenital malformation**	**1 (0.1)**	0 (0)	1 (0.2)	**-**	-	-
**Undetermined**	**79 (5.4)**	**46 (5.7)**	**33 (5.4)**	**15 (10)**	**8 (9.1)**	**7 (11.3)**

Overall, the commonest, most probable cause of death was birth asphyxia (43%) followed by neonatal infections (sepsis, meningitis, and pneumonia) (29.3%) and prematurity (22.2%). There were fewer neonates who died because of prematurity among those who died at a health facility compared to those who died at home (16.8% *vs* 26.3% respectively, p<0.0001). By contrast, the proportion of neonates who died of birth asphyxia was greater in those who died at a health facility compared to those who died at home (46.8 *vs* 40% respectively, p = 0.010). The second most probable cause of death was neonatal infections (36%), prematurity (31.3%) or birth asphyxia (22.7%).

**Fig 3:**
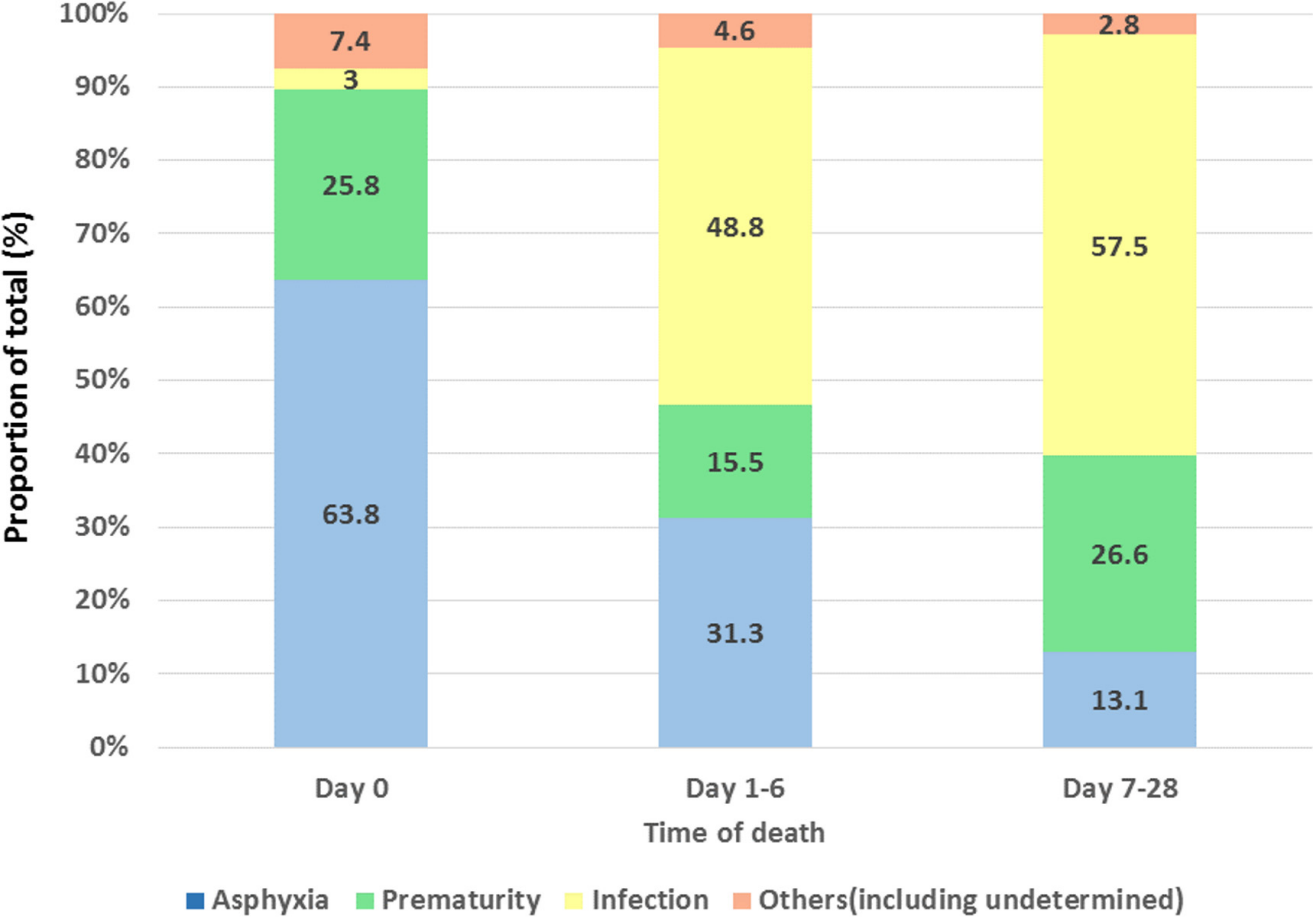
Main cause of death for early (first day, first week) and late (second and third week) neonatal deaths (% of all neonates in age group).

Asphyxia was the commonest cause of death (63.8%) for babies who died in the first 24 hours. For all other times, infection was the most likely cause of death (Fig 3). This trend was similar for both neonates who died at home or at a health facility.

For babies who died as a results of ‘other’ reasons (n = 60), even after review by the researchers of the information available, it was not possible to establish a likely cause of death (mainly as a results of insufficient information) and these were added to the group ‘undetermined’ (n = 19)

The distribution of cause of death for early neonatal death (day 0 to 6 inclusive) was asphyxia (50.2%), infection (22.5%), prematurity (21.3%), and undetermined (6.0%). For late neonatal deaths (day 7 to 28 inclusive) this was infection (58.5%), prematurity (25.6%), asphyxia (12.2%), and undetermined (3.7%).

### Pattern of care seeking by parents and factors associated with neonatal death

**Fig 4:**
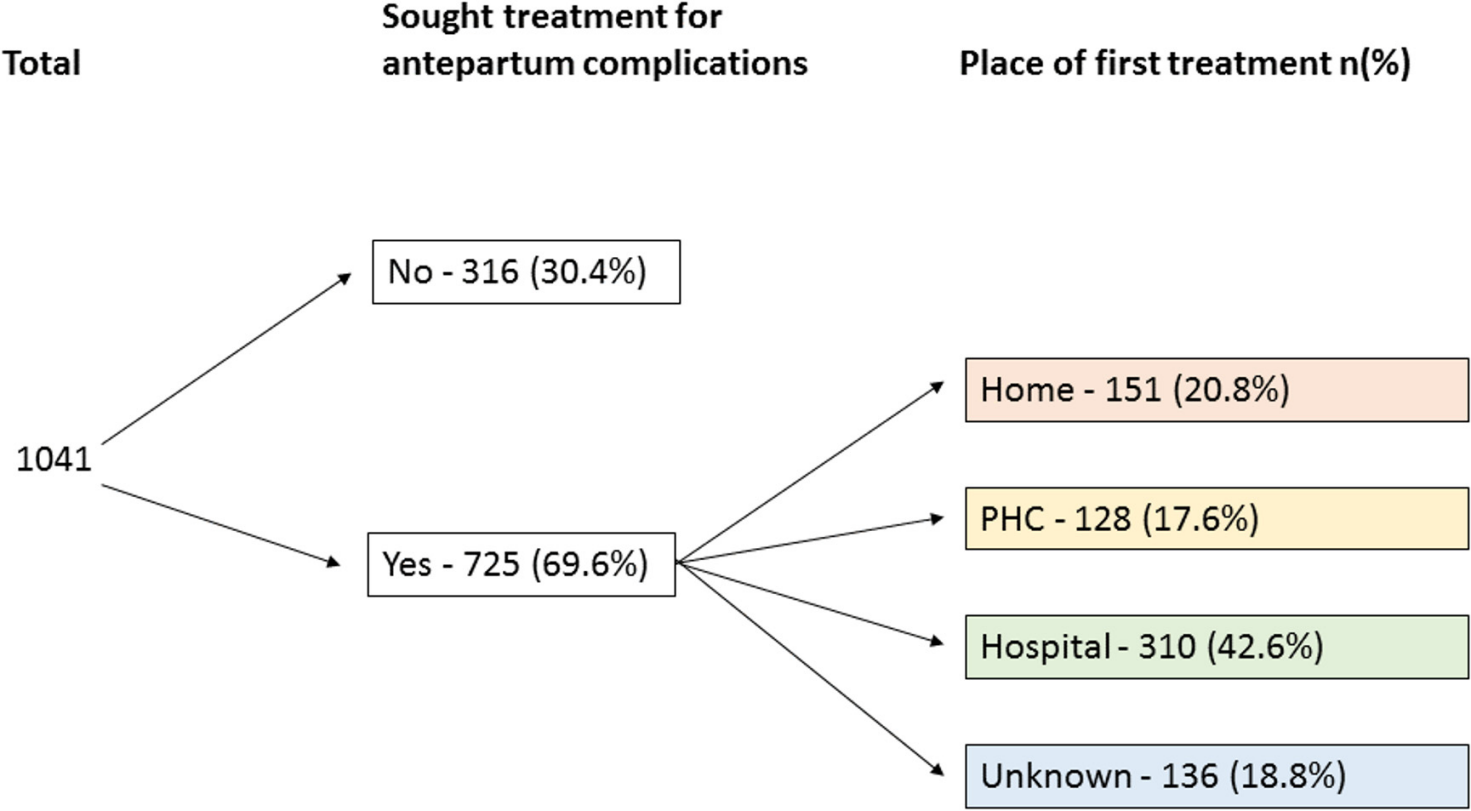
Pattern of care seeking by parents with a sick newborn who died.

The majority (68.3%) of parents sought care when the neonate was ill, of which, 69.5% sought care at a health facility (Fig 4). Among those who went to a hospital, 25.5% were transferred to another hospital to receive additional care. Thirty nine percent of parents sought care from more than one provider.

Mothers who received at least a primary education were 18% more likely to seek care (from a qualified or non-qualified provider) (binomial regression; RR 1.18, 95% CI 1.04–1.35) compared to mothers with no education. Parity was a determinant of care seeking; mothers who had two to four previous deliveries were 11% less likely to seek care (RR 0.89, 95% CI 0.82–0.96). However, this was no longer significant in women who had more than five deliveries. The mode and place of delivery was also an important determinant of care seeking. Parents of neonates born by vaginal delivery at a health facility and born by Cesarean Delivery were more likely to seek care (RR 1.48, 95% CI 1.37–1.60 and RR 1.52, 95% CI 1.42–1.54 respectively). Gender was associated with care seeking; for neonatal deaths among girls, parents were 13% less likely to have sought care compared to boys (RR 0.87, 95% CI 0.80–0.93). Similarly for neonatal deaths where babies were reported to have been small for dates, parents were 11% less likely to have sought care (RR 0.89, 95% CI 0.82–0.96). For babies who died as a results of asphyxia or prematurity, access to care was 18% and 28% less frequent when compared with neonates who died of infection (RR 0.82, 95% CI 0.76–0.88 and RR 0.72, 95% CI 0.64–0.80). Age of the mother, antenatal clinic attendance, complications during delivery, and spontaneous crying at birth were non-significant factors.

Among the 679 parents who sought care at a health facility, during the verbal autopsy information on the time needed to take the decision to seek care (Type 1 delay) was reported in 559 cases, reaching a facility (Type 2 delay) in 510 cases, and waiting time at the facility before care was received (Type 3 delay) in 425 cases. The decision to seek care was reported to have been made within one hour in 61% of cases, time to reach a facility was less than one hour in 51.4% of cases, and waiting time before receiving care was less than one hour in 87% of the cases (Table 3).

**Table 3:** Analysis of type of delay reported by parents of babies who accessed care at a health facility but whose baby died in the neonatal period (n = 679).

Type of delay	Total n (%)	Neonatal death at home n (%)	Neonatal death at heath facility n (%)
**Type 1 delay: Decision to seek care**	559	138	421
Within 1 hour	340 (61.0)	58 (42.0)	282 (67.0)
1–3 hours	120 (21.4)	36 (26.0)	84 (20.0)
4–6 hours	24 (4.2)	12 (8.7)	12 (2.8)
> 6 hours	75 (13.4)	32 (23.2)	43 (10.2)
**Type 2 delay: Time to reach facility**	510	123	387
Within 1 hour	262 (51.4)	44 (35.7)	218 (56.4)
1–2 hours	184 (36.1)	50 (40.6)	134 (34.6)
> 2 hours	64 (12.5)	29 (23.6)	35 (9.0)
**Type 3 delay: Waiting time at facility**	425	100	325
Within 1 hour	370 (87.0)	82 (82.0)	288 (88.6)
> 1 hour	55 (13.0)	18 (18.0)	37 (11.4)

Finally, we identified determinants of those care seeking at a health facility and compared them to those seeking care from traditional practitioners in the community. We found that the number of ANC visits, parity, and place of delivery were associated with seeking care at a health facility. Mothers who had attended for antenatal care four times or more were 30% more likely to seek care at a health facility rather than in the community (RR 1.30, 95% CI 1.12–1.52), delivery at a health facility and by Cesarean Delivery 103% (previous vaginal delivery), and 94% (previous caesarean section) more likely to seek care at a health facility (RR 2.03, 95% CI 1.83–2.25 and RR 1.94, 95% CI 1.75–2.16, respectively). Mothers who had five or more deliveries were, however, 26% less likely to seek care from a health facility (RR 0.74, 95% CI 0.57–0.96).

Among the 455 caregivers who did not seek care, the majority (60.5%) reported that they did not think that the condition of the neonate required health care at the time. In 37.6% of these cases (171 or 11.9% of total), the neonate died before any decision could be taken.

Other reasons for not seeking care included lack of transport (5.5%), lack of money for transport (12.5%), the nearest health facility was considered to be too far to travel (8.8%), the health facility was known to be closed (3.3%), or the healthcare provider was absent (1.5%). In 9.9% of the cases, the need for care was identified at night or during serious adverse weather conditions which prevented parents from seeking care. Finally, 2.6% of care givers considered the quality of care at the health facility to be poor.

## Discussion

### Main findings

Over a two-year period, all neonatal deaths that occurred in four districts in Bangladesh were identified and of these 1 in 5 was subjected to verbal autopsy. A neonatal mortality rate of 24.4 per 1,000 live births was obtained. Almost half of all babies (46.1%) died in the first 24 hours of life, 83.6% died in the first week of life (early neonatal deaths) and 16.4% in the second to fourth week (late neonatal deaths). This study shows that district level surveillance of all neonatal deaths can be introduced successfully and provides a population-based, contemporaneous, neonatal mortality rate as well as robust data on where and when during the first month of life these deaths occur.

Birth asphyxia was responsible for almost half of all deaths overall (43%) and the commonest identified cause of death in babies who died in the first 24 hours. Infections caused almost a third of deaths overall (29.3%) and was identified as the second cause of death overall for early neonatal deaths (22.5%) and the leading cause of late neonatal deaths (58.5%). Based on information obtained from parents during verbal autopsy, prematurity was a factor identified in almost a quarter (22.2%) of all deaths.

Additionally, this study demonstrates that for many babies care is available in principle with almost 68.3% of parents of babies who died having accessed care at a health facility with little or no reported delay in reaching or receiving that care and an additional 11.9% reporting that the baby had died before help could be sought most likely of acute problems. Among the minority of parents who did not seek care, the main reasons for this were failure to recognise danger signs early enough, transportation problems, or non-availability of a functioning health facility. Some parents also reported not accessing care because they considered the care available to be of poor quality.

### Strengths and limitations

More accurate data on number, cause of and factors contributing to neonatal deaths is needed. To the best of our knowledge this is one of the first and largest studies to introduce a perinatal mortality surveillance and review programme at population level in a low- or middle-income country. Given that the neonatal mortality rate obtained for the districts is very similar to that predicted using national estimates, it is likely that the vast majority of neonatal deaths were identified. With a large number of neonatal deaths it is not possible, nor probably necessary to review all deaths to understand the underlying cause and factors contributing to death; and we therefore reviewed 1 in 5 cases using verbal autopsy methodology.

The majority of the respondents were the mothers of babies who had died and were present at the moment of death. The interviews were held within a short period of time after the death, which reduced the risk of recall bias. We used the InterVA-4 computer automated model to determine likely cause of death. This allowed for a standardised interpretation of cause of death and is useful for processing large data sets. This study provides important information on the main (disease group level) causes of both early and late neonatal death and demonstrates that verbal autopsy at community level does allow for estimation of at least a likely cause of death for babies who died either at facility level or in the community. In addition, information on factors associated with death, health seeking behaviour, and experience of the quality of care received can be obtained by talking with the parents or family of the deceased child. For babies who died in a health facility, conducting facility-based death audit or review together with community-based review or verbal autopsy is recommended. This was not possible in this study but would likely have provided more detailed information and a more accurate distribution of cause of death possibly with a smaller proportion of ‘undetermined’.

In particular, obtaining reliable and accurate information on birth weight and gestational age at birth is difficult in most low and middle income settings and is generally not possible using standard verbal autopsy methods and tools.[12] Such information should be recorded at time of birth (for example on birth certificates) by skilled birth attendants where gestational age and birthweight have been established. For babies born in the community determination of gestational age at birth may be more difficult but it should be possible to weigh the baby and record birthweight. Globally, the distribution of cause of neonatal death overall includes prematurity (36%), birth asphyxia (23%) and infections (22%) [1]. If more detailed information regarding both gestational age and birthweight has been available in this study, more cases of prematurity might have been identified.

With regards to the relative importance of each cause of death, the fact that birth asphyxia was the main cause of death overall in this study is in line with other studies conducted in Bangladesh [13,14,15]. It must however be noted that the reported proportion of birth asphyxia was also high among babies who were born at a health facility in this study despite a higher rate of caesarean section compared to other studies [16]. By contrast, smaller studies have reported infection to be the most frequent overall cause of neonatal death in Bangladesh [17,18,19]. Our larger data set, coupled with information on time of death allows for disaggregation and a better documentation and comparison of leading causes of death in the first 24 hours, first week, and first month of life illustrating that infection is the most likely cause of death in babies who survive the first day of life. The difference in findings could theoretically be related to the quality of management of neonatal infections. If infection is recognised early, managed properly at either facility or community level, and as a result is less likely to result in death, the relative importance of other causes increases. This requires further study, we do not have enough evidence to confirm that the treatment of infections in the districts where this study took place was better than in other parts of the country. This will also need to be monitored over time and, with surveillance in place, could be considered as a measurable outcome to assess effectiveness of community-based management of neonatal infection

### Interpretation in light of other studies

Identifying factors that determine care seeking behaviour of parents of sick neonates is important. Herbert and colleagues highlighted the lack of representative data on this issue from South Asia in a recent systematic review [20]. In this study from Bangladesh, educated mothers and those who had previously delivered in a health facility were more likely to seek care in general (from both informal and formal healthcare providers). In contrast, we found that mothers with two to four previous births, being a girl baby, being small for dates or premature, and mothers whose babies had died of birth asphyxia were less likely to have accessed care before the baby died.

Shah et al studied the pattern of care seeking for premature neonates in Bangladesh and showed that the majority of parents were reluctant to seek care and, when they did so, they preferred non-trained community practitioners [21]. A proposed explanation for this was that small babies are perceived to be more vulnerable by parents and therefore they should receive gentle care in the community rather than parenteral treatment in a health facility which is perceived to be more aggressive. An earlier study by Darmstadt et al, reported that preference was generally given to seeking care from unqualified practitioners in first instance [15].

It has been suggested previously that multiparous women may be less likely to access care earlier as they may be considered to be more knowledgeable and experienced with childhood illness [22]. However, in our study, 44% of neonates who died were from multiparous women indicating that this is an important group to target when it comes to ensuring parents recognise the sick baby and access care as soon as possible.

In terms of selection of the first point of care, almost 70% of all parents had accessed a hospital which was either a District Hospital, which is, in principle, equipped with a special neonatal care unit or a Medical College which can offer advanced care including ventilation support. Despite this, the neonatal mortality rate in the four districts at the time of the study was high suggesting that even though health care was available and accessed, the quality of care for sick neonates was insufficient to prevent at least some of these deaths.

In order to reduce neonatal mortality, the quality of care at both primary and secondary level will need to be re-assessed and improved. There is still a need to continue to alert parents to the danger signs and the need to seek early care for sick babies and perhaps especially those who already have a family, low birth weight babies, and/or babies born prematurely. At the same time, improving the capacity of primary healthcare workers to recognise and effectively manage asphyxia and infection in the neonate is crucial. Providing simplified but effective antibiotic treatment (consisting essentially of oral antibiotics combined with just two days of parenteral antibiotics) can be expected to lead to improved outcomes as recommended after the recent trial on simplified antibiotic regimes for neonatal infections [23]. Although we note that in this study, the majority of parents did not report experiencing significant delays in accessing or receiving care, the implementation of a free of charge transportation scheme for sick neonates, is likely to contribute to the reduction of neonatal mortality where access to care is a problem or referral needs to be expedited.

The improvement of care seeking is of limited use if the quality of care provided either at the community or health facility level is poor. It is crucial that the quality of evidence-based care packages such as skilled birth attendance, emergency obstetric care, basic neonatal care and care of the sick neonate are strengthened at all levels. Minimum available care packages have largely been defined but the components of these are still not available at all levels in many settings [24,25]. Standards and indicators to assess quality of care provided during pregnancy, birth, and in the postnatal period for mothers and babies have been developed and need to be adapted and adopted so that they can be used to monitor quality of care.

## Conclusion

Asphyxia and infection were the commonest causes of death. Most neonates who died had access to qualified care. Improving quality of care at both community and health facility level is needed if neonatal mortality is to be reduced and the new targets set for 2030 are to be reached.
